# Phylogenetic Relationships in the Miracle Berry Genus, *Synsepalum*, Sensu Lato, and Relatives (Sapotaceae)

**DOI:** 10.3390/plants14010041

**Published:** 2024-12-26

**Authors:** Daniel Potter, Mark Uleh

**Affiliations:** Department of Plant Sciences, College of Agricultural and Environmental Sciences, University of California, Davis, CA 95616, USA; mark_uleh@yahoo.ca

**Keywords:** Phylogeny, *Synsepalum*, *Englerophytum*, Miracle berry, nuclear DNA, Plastid DNA

## Abstract

*Synsepalum* and *Englerophytum* are two closely related genera of the sub-family Chrysophylloideae in the family Sapotaceae. It has been reported that the two genera are a monophyletic group, and their generic limitations are uncertain. *Synsepalum* is an economically important genus that includes the medicinally and culinarily important plant, -miracle berry, *S. dulcificum*. The phylogenetic relationships among the species are poorly understood. This study has been conducted to refine the phylogenetic relationships between and within the two genera utilizing nuclear and chloroplast DNA data. Bayesian analyses and Maximum likelihood of nuclear internal transcribed spacer (*ITS*) and plastid (*trnH-psbA*) sequences were used to reconstruct the phylogeny of the two genera. Phylogenetic trees generated for both regions (nuclear and plastid) resulted in the resolution of six clades. Four of the clades correspond to species in the genus *Synsepalum* and two clades include species of *Englerophytum*. The two clades of *Englerophytum* are nested within *Synsepalum* suggesting that the two genera are closely related and may not merit their current circumscription as distinct genera. Also, *Synsepalum* is confirmed to constitute more than one lineage suggesting it is not monophyletic in its current definition. Overall, the study suggests the need for a comprehensive evaluation of all species currently recognized in the two genera.

## 1. Introduction

*Synsepalum* (A. DC.) Daniell and *Englerophytum* K. Krause are two closely related genera of the sub-family Chrysophylloideae [1] in the family Sapotaceae. These two genera comprise 35 and 19 recognized species respectively and are predominantly distributed across West-Central tropical Africa [2]. Both genera share the frequent presence of stipules, usually 5-merous flower with the irregular presence of small staminodes, similar seeds, and embryos. They are however considered to be different genera due to the consistent striate brochidodromous venation and strong fusion of the filaments into a staminal tube found in species of *Englerophytum*, whereas in species of *Synsepalum* leaf venation tends to be eucamptodromous and the filaments are free. In their study of the *Synsepalum*-*Englerophytum* complex, [2] reported six lineages from combined data from nuclear DNA, chloroplast DNA and morphology analyzed using parsimony. The views of [2] are however different from the previously obtained results from [3,4] in which the two genera formed a single heterogeneous clade where species of *Synsepalum* genus were grouped within species of *Englerophytum*. The differences between the two genera are a call for concern as the decision to either merge the two genera or separate them is yet to be reached. Also, [5] in their multi-gene phylogeny study, found support for the monophyly of the *Synsepalum*-*Englerophytum* clade but did not sample either genus extensively, only very limited sampling of the two genera.

### 1.1. History of the Classification of Synsepalum

*Synsepalum* has undergone several taxonomic changes throughout history as new species have been discovered. It is comprised of trees and shrubs native to tropical lowland areas of Africa. It was described in 1852 and currently consists of about 35 species [2], including the very popular miracle berry plant, *S. dulcificum* (Schumach. & Thonn.) Daniell which is the type species on which the genus is based. Like the genus *Diploon* Cronquist, *Synsepalum* has glabrous staminodes and imbricate to valvate corolla lobes [6]. A very common feature in the genus is their fused sepals, a character that gave the name to the genus. *Synsepalum* can also be characterized by its long spreading corolla lobes and large stipules [6]. The current 35 recognized species in the genus are a combination of species from previously recognized smaller genera, including *Afrosersalisia* A.Chev., *Pachystela* Radlk, *Vincentella* Pierre, *Synsepalum*, and *Tulestea* Aubrév. & Pellegr.

Previous generic classifications were considered unsatisfactory and therefore the genera were united under *Synsepalum* [7]. The small genera were merged using overlapping characters to form the currently recognized genus. The combination of the following characters was used to describe *Synsepalum*: frequent occurrence of large stipules, eucamptodromous venation, 5-merous flowers, corolla nearly always rotate, cyathiform or shortly tubular with wide-spreading lobes, corolla lobe aestivation imbricate or induplicate valvate, stamens fixed at or near the top of the corolla tube, exserted with well-developed filaments. The seed is broad and not laterally compressed, with a broad adaxial scar that sometimes extends to cover most of the surface. The embryo has plano-convex cotyledons and endosperm is known to be generally absent in the genus. Due to the inconsistency in the characters of the small genera that were merged, species in the genus are often individually very distinct. This has complicated the taxonomic revision of the genus and caused many synonyms to have emerged. With the emergence of molecular technique, the lumping of these genera to form the genus *Synsepalum* has been disputed by many authors as the conclusion was purely based on morphological characters.

### 1.2. History of the Classification of Englerophytum

*Englerophytum* K. Krause was described as a genus [8], with *Englerophytum stelechanthum as* the type species. Five species were added to the genus [9,10], two of which were newly described while the other three were products of new combinations of species previously classified in different genera. As opposed to the views of [11], who advocated for the distinct status of the genera *Englerophytum*, *Wildemaniodoxa* Aubrév. & Pellegr. and *Zeyherella* (Pierre ex Baill.) Aubrév. & Pellegr, [7] united the genera based on the fusion of their filaments and the number of floral parts. Although [7] considered *Synsepalum* to be closely related to *Englerophytum* because of the frequent presence of stipules, usually 5-merous flowers, irregular presence of small staminodes, and similar structure of seeds and embryo, he considered *Synsepalum* distinct genus from *Englerophytum*.

### 1.3. Genera Merged by Pennington 1991 and Their Current Taxonomic Status

To understand the *Synsepalum*-*Englerophytum* complex better, it is important to know the previous genera merged to form the complex. As already highlighted by [2], 16 genera were merged to form the two genera. Table 1 below shows the different genera, their type species, and their current taxonomic status.

### 1.4. Distribution of Species in the Synsepalum and Englerophytum Complex

**Figure 1:**
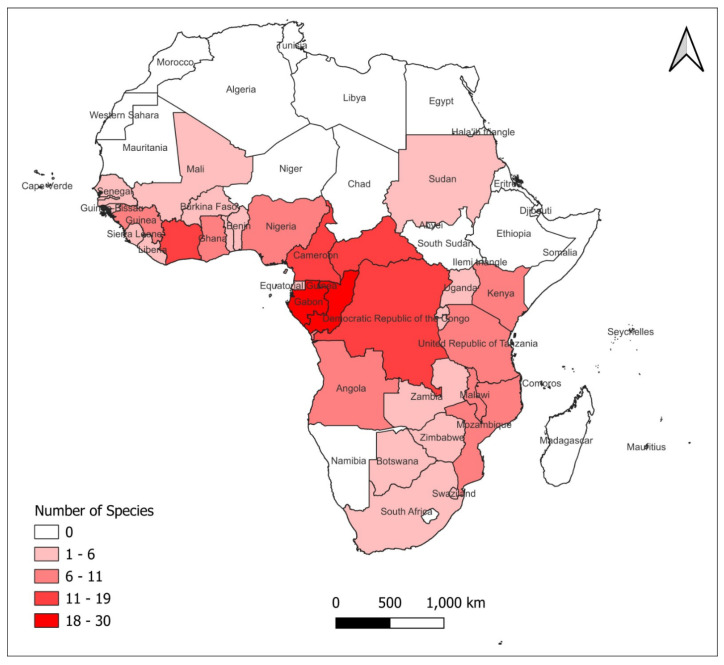
Map of Africa showing species distribution in *Synsepalum* and *Englerophytum*. Map designed using species distribution for the different countries.

Species of *Synsepalum* and *Englerophytum* are distributed across tropical Africa. While some are endemic to just one country like *S. aubrevillei* in Cote d’Ivoire, others have a wide range of distribution in different countries in tropical Africa. Also, as seen in Figure 1 below, many species in the genus *Englerophytum* are endemic to Gabon. Species in both genera are mostly trees and are mostly found in the wet tropic biomes, with few species found in the seasonally dry region.

### 1.5. Previous Phylogenetic Analysis

The Sapotaceae classification of [7] was purely morphological as that was the standard used then in reaching taxonomic conclusions. Nuclear DNA and plastid *trn*H-*psb*A were used by [2] to estimate phylogeny within the *Synsepalum*-*Englerophytum* clade. Their results do not support the classification by Pennington, and the species of the two focal genera of this study that they analyzed were resolved in a polytomy of six clades: two comprising the species of *Englerophytum* and four of *Synsepalum*. However, their result cannot be considered final due to incomplete sampling, as only 11 out of the 35 accepted species of *Synsepalum* and 8 out of the 19 species of *Englerophytum* were used for the study. They also recommended that more work is required before a comprehensive taxonomic conclusion about the clade can be reached.

Aside from the work of [2], there are no published reports on phylogenetic relationships within the *Synsepalum-Englerophytum* clade. In their studies of the *Synsepalum-Englerophytum* complex, [2] reported that four of the six lineages comprised *Synsepalum* species, and three out of the four lineages of *Synsepalum* corresponded to the smaller genera of the earlier generic classification by [9]. There are, however, some concerns with the lineages reported. Some of the lineages had just a single species, which was not the type species of the small, segregated genus (e.g., the type species of *Vincentella* was not included in the study). More species need to be investigated to better understand the phylogenetic relationships among species currently classified in *Synsepalum* and *Englerophytum* and to determine the number, names, and circumscriptions of genera that should be recognized in a phylogenetically based classification.

### 1.6. Significance of the Study

In general, plant phylogenetic studies provide a framework for understanding the fundamental processes of evolution and help in organizing the diverse plants of the earth in a way that will make sense to all. In the genus *Synsepalum*, although the presence of stipules and 5-merous flowers has been suggested as diagnostic characters for the genus, the presence of stipules is not consistent. They are missing in some species, (e.g., *S. dulcificum*); these may represent secondary losses, however. Phylogenetic analyses based on molecular data should make it possible to evaluate relationships among species in the group and compare them with the ancient generic concept. Moreover, not much has been done in resolving the divergent views of researchers on the merging of the small genera by Pennington to form *Synsepalum sensu lato*. This research proffers a solution to taxonomic problems in the *Synsepalum*-*Eglerophytum* complex.

## 2. Materials and Methods

### 2.1. Sampling of Taxa

It is generally believed that fresh materials from the field are more reliable for DNA extraction but due to the outbreak of the Covid-19 pandemic, getting to the field to sample materials was not an option to be explored for this study. Thus, materials for both *Synsepalum* and *Engleropytum* were mostly accessed from herbarium material. Materials were obtained as loans through the University of California Davis Herbarium (DAV). Samples were collected from Missouri Botanical Gardens (MO), New York Botanical Gardens (NY), Harvard University Herbarium (HUH) and The Conservatory and Botanical Garden of the city of Geneva (Conservatoire et Jardin botaniques de la Ville de Genève including many samples a researcher from G had graciously forwarded to us after obtaining permission from MusÌ©um National d’Histoire Naturelle Herbier National de Paris, France, P). A few other samples were collected in silica gel from people who grow them in their gardens. Leaf material sufficient for use in extracting DNA was removed from the herbarium samples. To avoid the destructive removal of leaf samples, leaves already placed in the fragmented packet in the herbarium sheets were first used. Where there were no leaves in the fragment packet, a single leaf was removed and used for the experiment. A total of 103 leaf samples were used for this study, comprising 43 from different herbaria in the United States (MO, NY, and HUH), 56 from Switzerland (G) and France (P), and four were fresh samples, see Supplementary Materials, Table S1.

### 2.2. Genomic Regions Selection

In this study, for the nuclear region, *ITS4* & *ITS5* primers were used while for the chloroplast regions, *trnH-psbA*, was used. The choices of the regions and primers were based on previous studies of the family *Sapotaceae*. Several researched articles on phylogenetic studies in *Sapotaceae* have shown that both *ITS* and *trnH-psbA* are excellent primers in the study of species relationships in the genus and family in general [4,12].

### 2.3. DNA Extraction

DNA was extracted from all the 103 samples. Two methods were used for grinding. Liquid nitrogen was applied to 20 mg of leaf tissue in a mortar and pestle and the leaf was ground to produce a fine paste. In some cases, about 20 mg of leaf tissue mixed with 20 mg of PVP was ground in two 30-s cycles in a BeadBug Mini Homogenizer Model D1030. DNA was extracted from ground leaves using the DNAeasy plant DNA extraction kit (Qiagen, Valencia, CA, USA). The extraction of DNA was according to the manufacturer’s instructions with slight modifications for some samples. Where DNA extracted using Qiagen did not provide good bands during PCR, CTAB was used to extract DNA.

### 2.4. Amplification and Sequencing

For nuclear DNA, 10 μM of the primers *ITS4* (TCCTCCGCTTATTGATATGC), *ITS5* (GGAAGTAAAAGTCGTAACAAGG) ([13]) was used. The reactions were carried out in 50 μL comprising of the master mix in the table below:



**Reaction**

**Volume (μL)**
1. DD water412. Coral load buffer53. DNA template24. DNTP15. Taq0.506. Forward primer0.257. Reverse primer0.258. Total50


Applied Biosystems 2720 thermal cycler made in Singapore was used. The thermal cycling profile was generally that suggested by the manufacturer: 95 °C for 5 min, followed by 35 cycles of 95 °C for 30 s, 50 °C for 30 s, 72 °C for 90 s, and additional cycle at 72 °C for 8 min.

For the chloroplast genome, 10 μM of trnH (ACTGCCTTGATCCACTTGGC), psbA (CGAAGCTCCATCTACAAATGG) [14], was used. The reactions were carried out in 25 μL comprising the master mix in the table below:



**Reaction**

**Volume (μL)**
1. DD water17.252. Ammonium (NH4)2.53. MGCL21.254. DNA template15. Forward primer0.756. Reverse primer0.757. DNTP0.58. BSA0.89. Taq0.210. Total25


The thermal cycling profile setting for the chloroplast region was 80 °C for 5 min, followed by 35 cycles of 95 °C for 1 min, 50 °C for 1 min, 62 °C for 5 min, and an additional cycle at 65 °C for 5 min at a ramp rate of 0.3 °C/s.

### 2.5. Gel Electrophoresis

The amplified fragments for both regions were controlled for their quality by electrophoresis. 1.8 g of powdered agarose gel was added to 100 mL of 1X TAE buffer. The mixture was shaken vigorously to ensure the agarose gel was completely immersed in the 1X TAE buffer. The mixture is heated in the microwave for 1 min or 90 s to ensure the agarose gel has completely melted. After heating, 1 μL of Sybrsafe DNA gel stain is added to the beaker containing the agarose gel, which is placed in a bath containing water for a few seconds until the beaker is cool enough to be handled with the hand using hand gloves. The gel solution was poured into a tray fitted with combs and allowed to stay for 20 min until it solidified. After solidification, the comb is removed, and the wells are loaded with PCR. The chamber containing the loaded DNA is connected to power at 76 KVA and allowed to run for 1 h. The gel is then visualized under UV light. Wells that produce bands are considered successful. The bands are excised using a razor blade. DNA was extracted from the bands and purified by application of a QIA quick PCR purification kit from Qiagen (Qiagen, Valencia, CA, USA).

### 2.6. Sequence Editing and Alignment

To obtain DNA sequences, extracted purified DNA from the gel was sent to the UC Davis sequencing center. For each direction of the primer, six micro-liters were used. Raw data from the facility were opened on Sequencher 5.4.6 (Gene Codes Corporation, Ann Arbor, MI, USA) which was used to assemble contigs and edit the sequences. The first nucleotides of each end of the sequences were trimmed until readable bases were obtained. After trimming up the sequences, BLAST searches were performed to ensure the results obtained were that of Sapotaceae. In cases of contamination, blast results give different plant families and in some cases insects. Whenever contamination was observed the experiment was repeated to be sure the right species was used for the research. Alignment was done using muscle in MEGA X. For the GenBank codes of sequences used in our phylogenetic analyses, see Supplementary Materials, Table S2.

### 2.7. Phylogenetic Analysis

The evolutionary history was inferred using the Bayesian Inference and Maximum Likelihood methods.

Bayesian analysis—The dataset was analyzed with Bayesian inference using the program MrBayes version 3.2.7a [15]. Sequence data were subjected to a general time reversal model including the estimation of invariant sites and assuming a discrete gamma distribution with six rate categories (GTR+I+G). The relative fit of various models of nucleotide substitution for the *ITS* region, chloroplast regions, and combined data set to identify the best model was examined. The best model was selected based on the Akaike Information Criterion (AIC). The Markov Chain Monte Carlo (MCMC) sampling, starting from random trees and priors, was run for 1,000,000 generations and every 100th tree was sampled. Four MCMC chains comprising three heated chains and a single cold chain were used in the analyses. Majority rule consensus trees and posterior probabilities for nodes were assembled from all post-burn-in sampled trees. Phylogenetic reconstructions were estimated after a couple of independent runs to confirm that they converged on similar stationary parameter estimates. For the combined data set, the data for each region were merged and aligned using muscle in MEGA before running on MrBayes.

Maximum Likelihood—For Maximum Likelihood estimation, different regions used different models. For the nuclear region, *ITS 4* and *ITS 5*, sequence alignment was performed using the muscle tool included in the MEGA 10 suite, and the Kimura-2 parameter model was applied. For the chloroplast region involving trnHpsbA primer, alignment was also performed using muscle, but the best model was the Hasegawa-Kishino model. For the combined data, alignments were performed using muscle while the best model was the Tamura 3-parameter model. The bootstrap consensus tree inferred from 1000 replicates in Maximum Likelihood, was taken to represent the evolutionary history of the taxa analyzed. The percentage of replicate trees in which the associated taxa clustered together in the bootstrap test is shown next to the branches in the trees generated.

## 3. Results

### 3.1. Amplification of Chloroplast and Nuclear DNA Regions

After extraction of DNA, poor bands were obtained during PCR for many of the species studied. This could be due to the old nature of the herbarium specimens used. Several primers were tried for the chloroplast region but for many of them, only very few specimens were amplified during PCR. The best result for chloroplast region was obtained from *trnH-psbA*. When samples were sent for sequencing, most of the results obtained for *trnH-psbA* were good for one direction, and rarely did we get good results for both directions. To protect the integrity of the results, only directions with clean DNA sequences were used for alignment and analysis. This problem with *trnH-psbA* that is reported here was also encountered by [2].

A higher number of successful PCR reactions were obtained for the nuclear *ITS* region than for the chloroplast regions, unlike the result reported by [2]. Sequences obtained for the *ITS* region ranged from 500 to 550 bp, while those for the chloroplast region were mostly below 500 bp. Although more sequences amplified for *ITS*, the tree generated for both nuclear and chloroplast regions were not significantly different, so the data were combined.

### 3.2. Combined Datasets

Clean reads that are suitable for analysis were obtained for the two primer sets used for this study, but more samples were amplified for the nuclear region. To combine the data, only samples that we got sequences for both regions were used. This is to ensure that none of the regions will influence the topology of the tree more than the other. Also, for ease of comparison of clades obtained from the combined dataset to clades from the separate regions.

### 3.3. Bayesian Inference

**Figure 2:**
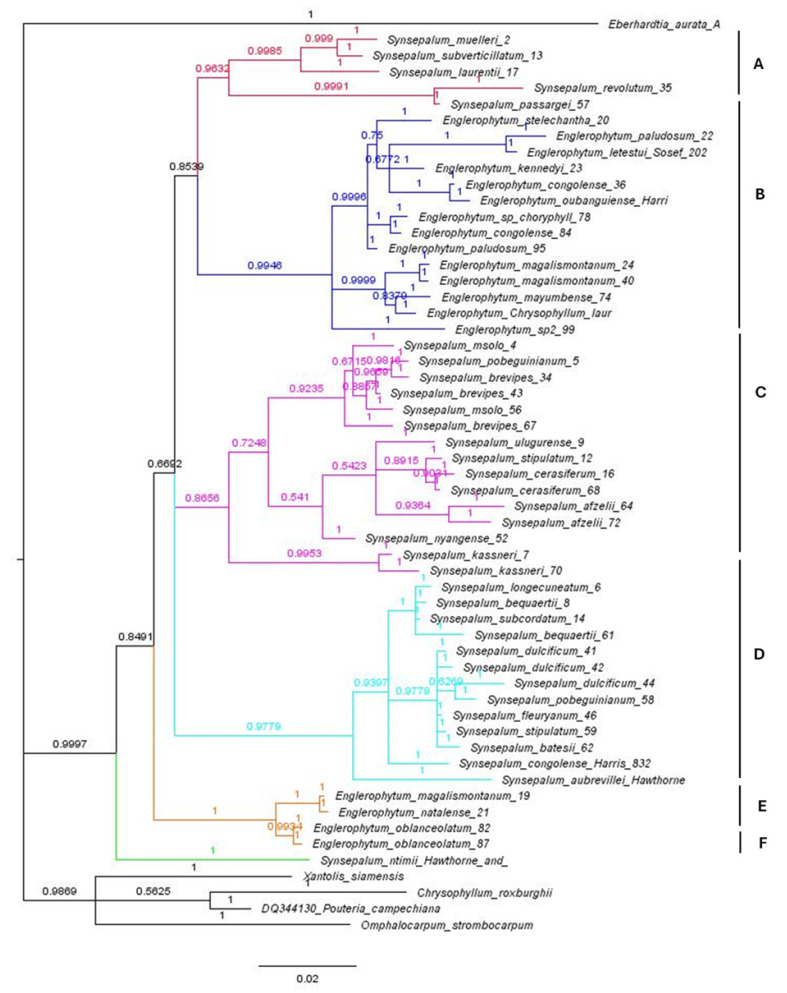
A 50% majority rule Bayesian consensus tree of the combined dataset revealing the different clades and their posterior probability.

Tree from combined dataset is shown below, Figure 2. The values of the posterior probabilities of the branches obtained are used as measures of branch support. Values below 0.95 are considered to have very low support. All the trees obtained from Bayesian inference and Maximum likelihood show a close relationship between *Englerophytum* and *Synsepalum*. Trees from separate analyses are available from corresponding author.

### 3.4. Monophyly of Synsepalum-Englerophytum

Tree topology from our Bayesian and Maximum likelihood analysis support the monophyly of a group including species of *Synsepalum* and *Englerophytum*. *Englerophytum* is nested within *Synsepalum* in all the phylogenetic trees obtained. Data analyzed resulted in six different clades. Two out of the six clades generated were exclusively for species in the genus *Englerophytum*, while four clades were species in the genus *Synsepalum*.

### 3.5. Monophyly of Synsepalum and Englrophytum

Neither *Synsepalum* nor Englerophytum were not resolved as monophyletic and some of the clades in the polytomy corresponded to the previously recognized genera by Aubréville. Three of the species previously grouped under the genus *Afrosersalisia* were used in this study namely *S. afzelli, S. cerasifera,* and *S. kassneri*. As seen in Figure 2, they belong to the same clade,—clade C. For the *Vincentella* genus, out of the four species, data were successfully obtained for three species. These include *S. muelleri*, *S. passargei*, and *S. revoluta*. None of the herbaria we loaned specimens from had *S. brenanii*. Just like the report for *Afrosersalisia*, the species are in the same clade, -clade A in Figure 2. The case is slightly different for the genus *Pachystella*. Out of the three species in *Pachystella*, two species (*S. msolo* and *S. brevipes*) are in the same clade, clade C, separating them from *S. subverticillatum*, which was found in clade A. For the genus *Synsepalum* sensu stricto, three species were previously recognized. These include *S. dulcificum*—the type species for the genus, *S. stipulatum*, and *S. subcordatum*. All three species were included in this study. In this group, all the species were in one clade, clade D. It is important to note, however, that these three species were not the only species found in the clade, other species formed the same clade with them.

## 4. Discussion

The ITS regions, *ITS 4* and *ITS 5* gave the best results for all the primers used in this experiment. Many of the herbarium specimens did not provide good bands in PCR for some chloroplast primers. For the *ITS* primers, PCR not only provided good bands for many of the species but also had good sequence data when the extracted DNA from the gel was sent for sequencing. The *ITS* region had better success as it amplified more species compared to the chloroplast region.

### 4.1. Clades

Only the combined data tree is used for discussion in this paper. Other trees generated from nuclear and chloroplast regions are available upon request from the corresponding author. The combined data tree is not very different from the trees produced by separate analyses of the nuclear *ITS* and chloroplast regions; in all the trees, the species are separated into six clades comprising two *Englerophytum* and four *Synsepalum*. Thus, the generic delimitations within the *Synsepalum*-*Englerophytum* clade remain unclear even when the data were combined. It is good to emphasize here again that the current delimitation of *Synsepalum* and *Englerophytum* as circumscribed by Pennington cannot be substantiated using molecular evidence.

Clade A

As shown in the tree, clade A consists of *S*. *revolutum*, *S*. *passargei*, *S*. *laurentii*, S. *muelleri*, and *S*. *subverticillatum*. Aside from *S. passargei*, they are mostly trees. Although there is no single morphological character that unites all the species, stipules are present for most of them. Also, they all have alternate leaves except for *S. passargei* its leaves are crowded at the branch end. This clade is very similar to the genus *Vincentella*, one of the previously recognized genera merged by Pennington. The only species in the genus that is missing is *S. brenani.*

Clade B and E comprise of species in the genus *Englerophytum*. The genus did not resolve as a monophyletic. This was also observed by [2].

Clade C

The clade comprises *S. afzelii*, *S. ulugurense*, *S. cerasiferum*, *S. stipulatum*, *S. pobeguinianum*, *S. brevipes*, *S. msolo*, and *S. kassneri*. There are species from the different genera found in this clade. While *S. afzelii* and *S. cerasiferum* are from the previous genus *Afrosersalisa*, *S. msolo* and *S. brevipes* are from the genus *Pachystella*. The two species present in this clade (*S. brevipes*, *S. msolo*) both have stipules. They both have seeds that are ellipsoid in shape.

Clade D

In the previous genera, *Synsepalum* in the strict sense had three species. This includes *S. dulcificum*, *S. stipulatum*, and *S. subcordatum*. All the species in *Synsepalum* in the strict sense are in clade D. The clade also contained other species.

Clade F

The only species in this clade is *S. ntimii*. Not only in the combined data tree that this species resulted in a different clade. Even in the *ITS* tree, the species is separated into a different clade, suggesting a possibility of having it as a distinct genus.

### 4.2. Implications for Synsepalum

Two principal issues are addressed in this work. One is the merging of the small genera recognized by [7], while the second issue is the divergent view of some authors on the *Synsepalum*-*Englerophytum* complex. The previously recognized small genera *Afrosersalisia, Vincentella*, *Pachystela*, *Synsepalum*, *Tulesta*. After the revision of *Sapotaceae* by Pennington in 1991, 19 more species have been added to the genus *Synsepalum* bringing the total to 35 species currently recognized. Some authors including [2,3,6] have called for the separation of *Synsepalum* from the previous small genera that were combined [7].

As seen in the combined data tree generated, some of the clades corresponded to the previous genera that were recognized by Aubréville. Data analysis using Bayesian inference and Maximum likelihood for *ITS* grouped all three species of *Synsepalum* sensu stricto (*S. dulcificum*, *S. subcordatum,* and *S. stipulatum*) used in this study into one clade. A dichotomous tree having species of *Synsepalum* in one clade and species of *Englerophytum* in another clade would have supported the monophyly of *Synsepalum,* however, the tree obtained from the nuclear, chloroplast region and combined dataset resulted in a phylogenetic separation of subgroups of species belonging to both genera. In the combined tree reported here, clades of *Englerophytum* are nested within *Synsepalum*. This consistent nesting of *Englerophytum* within *Synsepalum* further suggests that the genera are closely related. Leaf venation, presence or absence of stipules, inflorescence, fusion of sepals, stamens insertion, anther position, and number of ovaries tend to overlap in the previously merged genera. The overlapping morphological character and the molecular evidence seen in tree suggest very strongly that *Synsepalum sensu lato* should be reversed into small genera.

### 4.3. Implications for the Synsepalum and Englerophytum Complex

Molecular evidence obtained from this study and all other phylogenetic studies involving the species merged by Pennington and the newly described ones for both *Synsepalum* and *Englerophytum* shows that the two genera are not phylogenetically distinct from each other. Thus, the trees obtained in this research do not agree with the circumscription of the genera as defined by Pennington. All the trees obtained using MrBayes for Bayesian inference and MEGA X for Maximum likelihood show that *Englerophytum* is nested within *Synsepalum*.

It is important to state here that only a few morphological characters were used by [7] to separate *Englerophytum* from *Synsepalum*. *Synsepalum* has an eucamptodromous venation and *Englerophytum* has a brochidodromous venation and a strong fusion of the filament into a staminal tube. Although it is taxonomically correct, in some cases, to use few morphological characters to separate genera, this is not just the case with the *Englerophytum*-*Synsepalum* complex. The nesting of *Englerophytum* within *Synsepalum* in trees generated for both nuclear chloroplast regions indicates very strongly that the group do not merit distinct generic status.

## 5. Conclusions and Recommendation

In this study of the phylogenetic relationships of the *Synsepalum*-*Englerophytum* complex, neither of the two genera resolved as monophyletic. Although resolution of relationships among the major clades were only weakly to moderately supported, the clades of *Englerophytum* are nested within *Synsepalum* as seen in the combined tree. This shows that the morphological circumscription of the two genera does not align with the molecular evidence. We think that morphological character states that distinguish *Synsepalum* might be ancestral (symplesiomorphies) while the character states that distinguish *Englerophytum* may be independently derived (homoplasious) synapomorphies for each subclade.

The polytomy observed in the genus *Synsepalum* shows that the genus is not a single lineage. Some of the clades in the phylogenetic tree correspond to some of the small genera merged by Pennington. The need to resurrect some of the previously merged genera, like *Vincentella*, is undeniably evident in the result obtained in this work and that of [2]. The *Synsepalum*-*Englerophytum* complex certainly requires some formal taxonomic changes to align with molecular evidence, but such changes cannot be made until a broader sampling of all species currently recognized in the genera is collected for comprehensive morphological as well as molecular studies. The complete genome of *Synsepalum* has been published. If the complete genome of *Englerophytum* is published, it would serve as additional information relevant to making definitive and comprehensive conclusions on taxonomic circumscriptions within the *Englerophytum*-*Synsepalum* complex.

## Figures and Tables

**Table 1:** Genera merged by Pennington (1991) to form the current *Synsepalum*-*Engleropytum* complex.

Genus	Type Species	Status
*Englerophytum* K. Krause	*E. stelechantha*	*Englerophytum*
*Bequaertiodendron* De Wild.	*E. congolense*	*Englerophytum*
*Tisserantiodoxa* Aubrév. & Pellegr.	*T. oubanguiensis*	*Englerophytum*
*Zeyherella* (Pierre ex Baill.) Aubrév. & Pellegr.	*Z. magalismontana*	*Englerophytum*
*Wildemaniodoxa* Aubrév. & Pellegr.	*W. laurentii*	*Englerophytum*
*Neoboivinella* Aubrév. & Pellegr.	*N. natalensis*	*Englerophytum*
*Pseudoboivinella* Aubrév. & Pellegr.	*P. oblanceolata*	*Englerophytum*
*Synsepalum* (A.DC.) Daniell	*S. dulcificum*	*Synsepalum*
*Stironeurum* Radlk	*S. stipulatum*	*Synsepalum*
*Vincentella* Pierre	*V. densiflora* (=*S. revolutum*)	*Synsepalum*
*Bakeriella* Dubard	*B. revoluta*	*Synsepalum*
*Pachystela* Pierre ex Radlk.	*P. longistyla*	*Synsepalum*
*Pseudopachystela* Aubrév. & Pellegr.	*P. lastourvillensis*	*Synsepalum*
*Afrosersalisia* A. Chev.	*A. afzelii*	*Synsepalum*
*Rogeonella* A. Chev.	*R. chevalieri*	*Synsepalum*
*Tulestea* Aubrév. & Pellegr.	*T. gabonensis*	*Synsepalum*

## Data Availability

Data are contained within the article and Supplementary Materials.

## Supplementary Materials

The supporting information can be downloaded at: https://www.mdpi.com/article/10.3390/plants14010041/s1.
