# Supplementary material for: Phylogenetic Relationships in the Miracle Berry Genus, Synsepalum, Sensu Lato, and Relatives (Sapotaceae)
Source: Plants (Basel). 2024 Dec 26;14(1):41. doi: 10.3390/plants14010041 (PMC11723033; doi:10.3390/plants14010041)
Supplement: Supplementary file 1 [file plants-14-00041-s001.zip › plants-3357528-supplementary.pdf]

Table S1: Species used for the study collected within the United States.

S/NO	Species name	Collector(s) Name	Collector number	Herbarium	GenBank ID ITS4&5	GenBank ID trnHpsbA
1	*E. magalismontanum*	D.A McCallum	1074	MO	PQ724347	PQ723341
2.	*S. longecuneatum*	Gordon McPherson	16736	MO	PQ724338	PQ723330
3.	*S. bequaertii*	Lee White	1115	MO	PQ724340	NA
5.	*S. cerasiferum*	Gordon McPherson	21391	MO	PQ724345	PQ723333
7.	*E. congolense*	Terese Butler Hart	1336	MO	PQ724355	PQ723340
8.	*S. subverticillatum*	Lenin Festo	2760	MO	PQ724343	NA
9.	*S. stipulatum*	Roy E. Gereau	7596	MO	PQ724342	PQ723331
10.	*S. subcordatum*	Terese Butler Hart	1092	MO	PQ724344	PQ723332
11.	*S. revolutum*	Thomas D.W	7998	MO	PQ724354	NA
13.	*S. muelleri*	J.D & E.G Chapman	9339	MO	PQ724335	NA
16.	*S. laurentii*	David J. Harris	2770	MO	PQ724346	PQ723334
18.	*S. ulugurense*	Moses A. Mwangoka	6515	MO	PQ724341	NA
19.	*S. brevipes*	M.Merello	1603	MO	PQ724353	NA
20.	*S. kaessneri*	C. J. Kayombo	2962	MO	PQ724339	NA
22.	*E. paludosum*	Ehoarn Bidault	1728	MO	PQ724350	PQ723337
24.	*E. stelechantha*	J.J. Wieringa	2321	MO	PQ724348	PQ723335
25.	*S. pobeguniana*	Chris S. Duvall	424	MO	PQ724337	NA
28.	*E. kennedyi*	J. Nemba	571	MO	PQ724351	PQ723338
32.	*E. magalismontanum*	E. Kaaya	2559	MO	PQ724352	PQ723339
33.	*E. natalense*	O.A. Kibure	1453	MO	PQ724349	PQ723336
34.	*S. msolo*	M.A. Mwangoka	216	MO	PQ724336	PQ723329
38.	*E. magalismontanum*	R. Brand	49	NY	PQ724356	NA
44.	*S. dulcificum*			Silica gel	PQ724357	PQ723342
45.	*S. dulcificum*			Silica gel	PQ724358	PQ723343
46.	*S. brevipes*			Silica gel	PQ724359	PQ723344

S/NO.	Species name	Species number	Herbarium	GenBank ID ITS4&5	GenBank ID trnHpsbA
4.	*E. parludosum*-95	600418157	G	PQ724378	NA
12.	*E. oblanceolatum*-87	600412472	G	PQ724377	PQ723352
15	*E. congolense*-84	MO3701061	G	PQ724376	NA
17.	*E. oblanceolatum*-82	600412473	G	PQ724375	PQ723351
21.	*E. chrysophyll sp.*-78	600412478	G	PQ724374	PQ723350
25.	*E. mayubense*-74	600412480	G	PQ724373	NA
27.	*S. afzelii*-72	600412443	G	PQ724372	NA
29.	*S. kaessneri*-70	600412422	G	PQ724371	NA
31.	*S. cerasiferum*-68	600412437	G	PQ724370	NA
32.	*S. brevipes*-67	600412439	G	PQ724369	NA
35.	*S. afzelii*-64	600412419	G	PQ724368	PQ723349
37.	*S. batesii*-62	600412441	G	PQ724367	NA
38.	*S. bequaertii*-61	600412440	G	PQ724366	NA
40.	*S. stipulatum*-59	600412464	G	PQ724365	PQ723348
41.	*S. pobeguinianum*-58	600412428	G	PQ724364	PQ723347
42.	*S. passargai*-57	600412426	G	PQ724363	NA
43.	*S. msolo*-56	600412424	G	PQ724362	PQ723346
47.	*S. nyangense*-52	600412431	G		PQ723345
	E. sp.2-99	600418336	G	PQ724379	PQ723353
53.	*S. fleuryanum*-46	600412433	G	PQ724361	NA

Table S2: Species collected outside the United States for this study

Table S3. List of species sequence borrowed from previous studies (Borg *et al* 2019)

S/NO.	**Species Name**	GenBank ID ITS	GenBank ID trnHpsbA
1.	*Synsepalum aubrevillei*	PQ722258	PQ722929
**2.**	*S. congolense*	PQ722260	PQ722931
**3.**	*S. ntimii*	PQ722272	PQ722943
**4.**	*Englerophytum oubanguiense*	PQ722238	PQ722907
**5.**	*E. letestui*	PQ722240	PQ722911

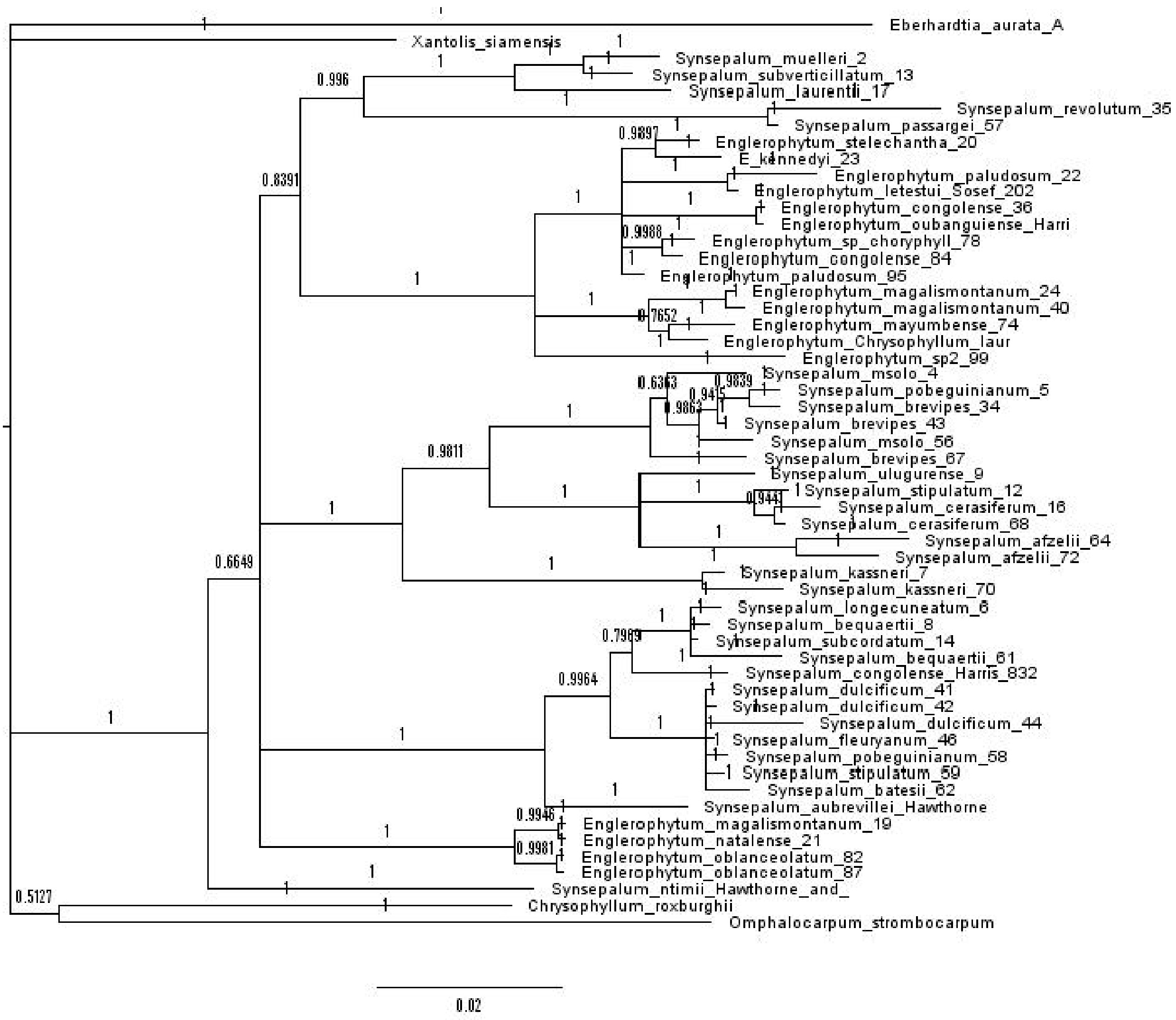

Figure. ITS Tree

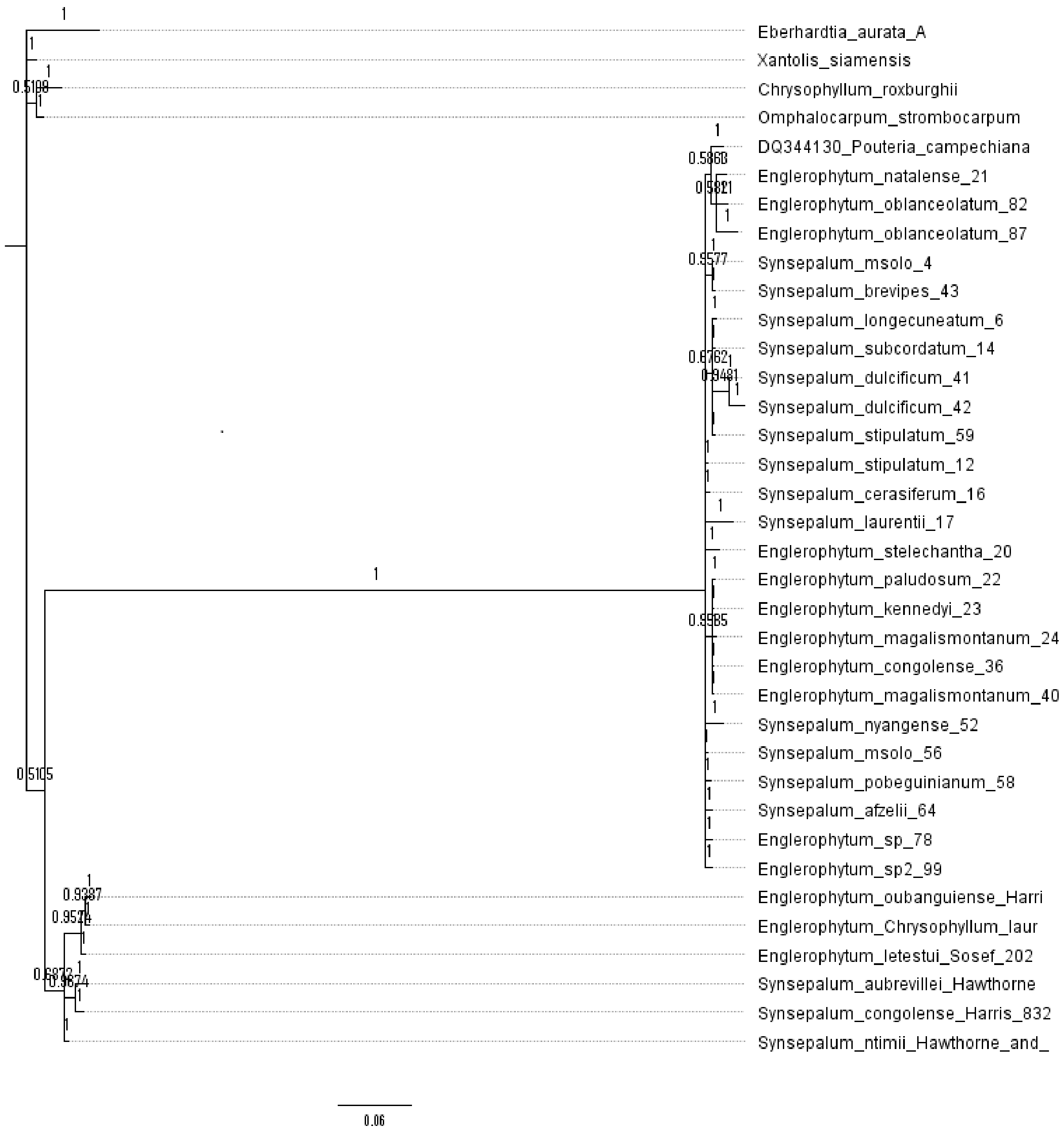

trnHpsbA tree
